# Distribution, diversity, mesonotal morphology, gallery architecture, and queen physogastry of the termite genus *Calcaritermes* (Isoptera, Kalotermitidae)

**DOI:** 10.3897/zookeys.148.1505

**Published:** 2011-11-21

**Authors:** Rudolf H. Scheffrahn

**Affiliations:** 1 Fort Lauderdale Research and Education Center, University of Florida, 3205 College Avenue, Davie, FL 33314 U.S.A.

**Keywords:** Neotropical distribution, species synonymy, field photography, taxonomic soldier key, mesonotal rasp, microbial symbiosis, queen physogastry, eggs

## Abstract

An updated New World distribution of the genus *Calcaritermes* is given along with photographs and a key to the New World species outside Mexico. *Calcaritermes recessifrons* is found to be a junior synonym of *Calcaritermes nigriceps*. Except for *Calcaritermes temnocephalus*, pseudergates of the other seven studied *Calcaritermes* species possess a mesonotal rasp. The rasps suggest a role in propagation of microbes on gallery surfaces and microbial infusion below the wood surface. *Calcaritermes temoncephalus* is shown to have an unusually large physogastric queens for a kalotermitid and several species produce large eggs.

## Introduction

In his monumental revision of the family Kalotermitidae, Krishna (1961) formed the current taxonomic definition of the termite genus *Calcaritermes* (Snyder 1925). Krishna (1961) separated *Calcaritermes* from all other genera by the diagnostic enlargement of the outer spine (“spur” *sensu* Snyder 1925b) of the fore tibia relative to the other two tibial spines. Soldiers also possess a dark, rather smooth, and cylindrical head capsule. *Calcaritermes* is a basal group within the Kalotermitidae (Legendre et al. 2008) and is not closely related to the sympatric *Cryptotermes* which also possess phragmotic dark-headed soldiers. Krishna 1961, however, could not morphologically distinguish *Calcaritermes* alates from those of the genus *Glyptotermes*. Even so, Emerson (1969) described *Calcaritermes vetus* from a fossilized alate in amber collected in the Simojovel region of Chiapas, Mexico. Emerson based his generic assignment on the similarity of the fossil to that of *Calcaritermes temnocephalus* and its range in southeastern Mexico. The most recent review of *Calcaritermes* distribution was also provided by Emerson (1969).

As with most non-pest termite genera, details of the ecology and bionomics of the *Calcaritermes* are completely unknown. Almost all that is published about *Calcaritermes* relates to identification of preserved specimens for faunal surveys (e.g. Scheffrahn et al. 2005 and part of this paper). The only research involving *Calcaritermes* biology stems from two studies: one of their protist gut fauna (Gile et al. 2010) and the other of alate flight in forest canopy (Bourguignon et al. 2009).

In the current paper, the New World distribution and diversity of *Calcaritermes* is revised based on material in the University of Florida collection. I use field photography to show the live habitus of castes of seven *Calcaritermes* species and depict eight soldiers using montage photography of preserved material. I also reassess the mesonotal “rasp” of pseudergate castes of *Calcaritermes* and provide an example of extreme queen physogastry in the Kalotermitidae. Finally, I describe the atypical feeding galleries of this genus and hypothesize a relationship between gallery architecture and the mesonotal rasp in terms of microbial symbiosis.

## Material and methods

**Figure 1.**
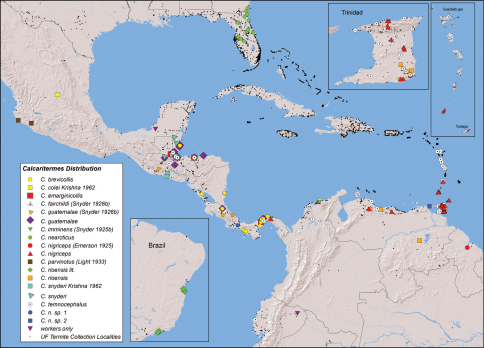
*Calcaritermes* distribution in the New World. Species and junior synonym names followed by citations or by “lit” are mapped from citation data only. All other species localities are mapped from records of the University of Florida Termite Collection.

A total of 214 colony samples of *Calcaritermes* from 122 localities (Fig. 1) were collected between 1996 and 2010 and identified by the author from original descriptions and comparisons. These samples are included in the University of Florida (UF) Termite Collection, Fort Lauderdale Research and Education Center, Davie, Florida. This collection houses over 34,000 samples, mostly from the Caribbean Basin, which the author and his colleagues have amassed since 1986. The findings herein are a direct result of field observations made while collecting *Calcaritermes* during various survey expeditions.

**Figure 2.**
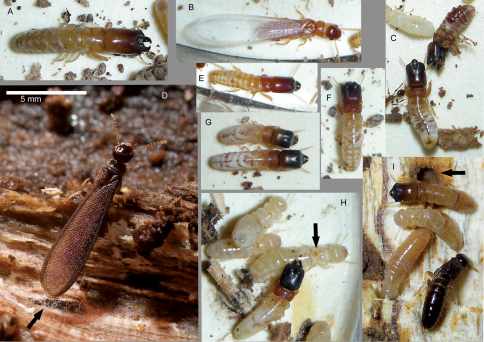
Photographs of live *Calcaritermes* specimens taken during collection. **A** Soldier *of C. guatemalae*, Honduras **B** Alate of *Calcaritermes temnocephalus*, Venezuela **C** Soldiers of *Calcaritermes snyderi* Honduras **D** Alate of *Calcaritermes guatemalae*, Belize (arrow denotes fungal hyphae growing in gallery) **E** Soldier of *Calcaritermes temnocephalus*, Venezuela **F** Soldier of *Calcaritermes brevicollis*, Colombia **G** Two soldiers from the same colony of *Calcaritermes nigriceps*, Colombia **H** Soldier and pseudergates of *Calcaritermes rioensis*, Venezuela (arrow denotes mesonotal rasp of pseudergate) **I** Soldier, dealate, and pseudergates of *Calcaritermes nearcticus*, Florida (arrow denotes fungal staining around gallery). All images to same scale.

**Figure 3.**
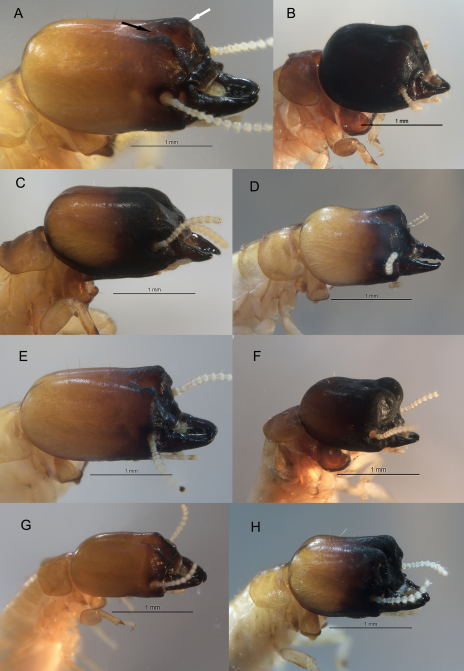
Oblique view of Calcaritermes soldiers from the University of Florida collection. **A** *Calcaritermes guatemalae*, Honduras (black arrow shows orientation of frontal furrow, white arrow points to frontal lobe) **B** *Calcaritermes rioensis*, Trinidad. **C** *Calcaritermes nigriceps*, Trinidad **D** *Calcaritermes nearcticus*, Florida **E** *Calcaritermes emarginicollis* Honduras **F** *Calcaritermes brevicollis*, Nicaragua **G** *Calcaritermes temnocephalus*, Guatemala **H** *Calcaritermes snyderi*, Guatemala. Enlarge outer tibial spines visible in B and G. Photos to same scale.

**Figure 4.**
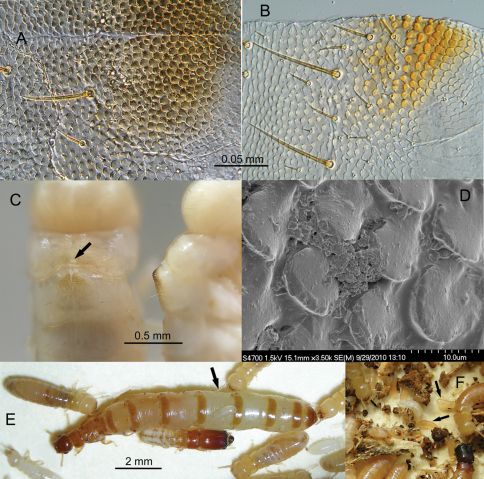
**A** Light micrograph of partial mesonotal rasp of *Calcaritermes nearcticus* pseudergate from an ethanol-preserved specimen. Mid-line is horizontal near top of figure **B** Micrograph of half of mesonotal rasp of *Calcaritermes brevicollis* pseudergate. Rasp is separated at the mid-line **C** Dorsal and lateral view of rasps on *Calcaritermes brevicollis* pseudergates (arrow denotes concavity of posterior margin of pronotum to accommodate elevation of the rasp) **D** SEM of rasp of *Calcaritermes nearcticus* from an unrinsed, freshly prepared specimen **E** Physogastric queen and other castes of *Calcaritermes temnocephalus* (arrow denotes egg on queen dorsum) **F** Eggs (arrows amongst nest debris) of *Calcaritermes brevicollis*, Colombia.

**Figure 5.**
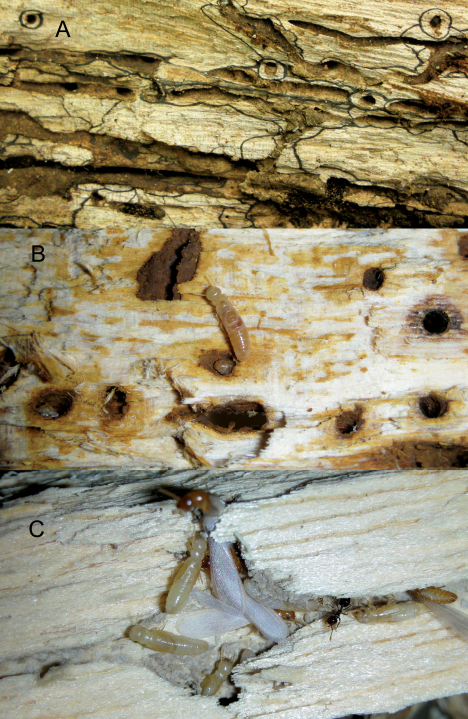
**A** *Calcaritermes nigriceps* galleries exposed in Colombia **B** *Calcaritermes nearcticus* galleries in oak wood, Florida **C** *Calcaritermes temnocephalus* galleries in Venezuela. Images not to same scale.

Field photographs (Figs 2, 4E, 4F, and 5) were taken with a Nikon Coolpix S7c digital camera set to macro and flash mode. Specimens were usually photographed in a 5.5 cm dia. plastic Petri dish bottom lined with manila folder cardboard although natural substrate (Figs 3D and 3I) was sometimes suitable. Figures 3 and 4C were taken as multilayer montages using a Leica M205C stereomicroscope controlled by Leica Application Suite version 3 software. Montage specimens were taken from 85% ethanol and suspended in a pool of Purell® Hand Sanitizer to position the specimens in a transparent plastic Petri dish. Mesonotal rasps (Figs 4A and B) were slide-mounted with PVA mounting medium (BioQuip Products, Inc) and photographed with an Olympus BH-2 compound microscope fitted with phase contrast optics. Figure 3D was taken of a pseudergate that was freshly killed by desiccation and photographed with a Hitachi 4700 FESEM scanning electron microscope at 3-5 kV.

## Results and discussion

### Distribution

*Calcaritermes* is primarily a neotropical genus with the exceptions of a relic nearctic species, *Calcaritermes nearcticus* Snyder, 1933, found from central and northeastern Florida to southeastern Georgia (Scheffrahn et al. 2001) and an anomalous indomalaysian congener, *Calcaritermes krishnai* (Maiti and Chakraborty) known from Great Nicobar Island (Roonwal and Chhotani 1989) and Papua New Guinea (Y. Roisin, unpublished data). The current New World distribution of *Calcaritermes* is given in Fig. 1. Literature localities in Fig. 1 include *Calcaritermes colei* Krishna from San Luis Potosi, Mexicoand *Calcaritermes snyderi* Krishna from El Salvador (Krishna 1962), *Calcaritermes imminens* from Colombia (Snyder 1925b), *Calcaritermes parvinotus* Light from Colima, Mexico (Light 1933) and from Chamela, Mexico (Nickle and Collins 1988) and *Calcaritermes rioensis* from Brazil (Krishna 1962, Reis and Cancello 2007). Emerson’s 1969 localities for *Calcaritermes guatemalae* (Tabasco region of Mexico) and *Calcaritermes nigriceps* (central Colombia) not mapped in Fig. 1 because they were deemed too vague.

### Nomenclatural revisions

Krishna (1962) redescribed *Calcaritermes temnocephalus* (Silvestri 1901) from types collected in Venezuela (Silvestri 1903) and additional material from Trinidad. The type locality, Las Trincheras (10.31, -68.09), Carabobo State, is in the vicinity of Caracas where Frederik Vilhelm August Meinert collected insects in 1891 (Reuter 1904) of which the termites were studied by (Silvestri (1901, 1903). In 2008, we collected all castes of a *Calcaritermes* sp. at P.N. San Sebastián, Carabobo, Venezuela (10.402, -68.000, elev. 105 m). Our material matched Krishna’s 1962 redescription of *Calcaritermes temnocephalus* and substantiates our earlier synonymy that *Calcaritermes fairchildi* is a junior synonym of *Calcaritermes temnocephalus* (Scheffrahn et al. in press). Specifically, the description of *Calcaritermes fairchildi* (=*thompsonae*) (Snyder 1926b, 1926c) from Costa Rica (Fig. 1) also compares favorably with our Venezuela sample. I have compared 50 colony series of *Calcaritermes temnocephalus* from Guadeloupe to Ecuador and Belize. *Calcaritermes temnocephalus* is unique among congeners in the UF collection (species shown in Fig. 3) because pseudergate castes do not have a mesanotal rasp and also lack the concavity of the posterior margin of the pronotum (Figs 4E, 5C). Imagos of *Calcaritermes temnocephalus* are unique among those described in the genus in that they are orange-brown in body coloration and have hyaline wings (Fig. 2B, 5C). The next lightest imago is *Calcaritermes brevicollis* with a medium brown dorsal coloration and lightly pigmented wings. All eight other described *Calcaritermes* imagos are dark brown to blackish and have smoky wings (e.g., Fig. 2D, 2I).

Snyder (1925b) described *Calcaritermes recessifrons* from one soldier and a series of alates (type locality Cincinnati, 11.10, -74.08, Fig. 1) collected by W. M. Mann during his expedition to Colombia. In 2009, we surveyed termites near the type locality for *Calcaritermes recessifrons* (“above” Minca 11.126, -74.120, elev. 712 m) and collected several colony samples of *Calcaritermes* there. Our material matched Snyder’s description of *Calcaritermes ressesifrons*. The description of *Calcaritermes nigriceps* (Emerson 1925) from British Guiana (Fig. 1, now Guyana) also compared favorably with our sample. Further comparison of *Calcaritermes* specimens collected from Grenada to Panama (Fig. 1) confirmed that *Calcaritermes recessifrons* is a junior synonym of *Calcaritermes nigriceps* as previously reported by Scheffrahn et al. (in press). *Calcaritermes nigriceps* soldiers are unique among congeners in the UF collection as the frontal furrow is shallow and unsculptured (Fig. 3C).Cincinnati, Colombia, is also the type locality of *Calcaritermes imminens* (Snyder 1925b); however, we were unable to collect this distinctive medium-sized species in which the soldier has an overhanging frons. Table 1 lists the current New World species of *Calcaritermes* and their type localities.

**Table:** Table 1. Revised New World list of *Calcaritermes* Snyder, 1925 and type localities.

*Calcaritermes brevicollis* (Banks 1918)	Panama*
*Calcaritermes colei*Krishna 1962	San Luis Potosi
*Calcaritermes emarginicollis* (Snyder 1926a)	Rio Chinilla, Canal Zone, Panama*
*Calcaritermes guatemalae* (Snyder 1926b)	Mixco, Guatemala*
*Calcaritermes imminens* (Snyder 1925b)	Cincinnati, Colombia
*Calcaritermes nearcticus* (Snyder 1933)	Clay County, Florida*
*Calcaritermes nigriceps* (Emerson 1925)	Kartabo, Guyana*
*Calcaritermes parvinotus* (Light 1933)	Colima, Mexico
*Calcaritermes rioensis*Krishna 1962	Ihla Grande, Rio de Janeiro, Brazil*
*Calcaritermes snyderi*Krishna 1962	Volcan de Santa Ana, El Salvador*
*Calcaritermes temnocephalus* (Silvestri 1901)	Las Trincheras,Venezuela (see text)*
Undescribed sp. 1	Boquette, Panama*
Undescribed sp. 2	Paria Pennisula,Venezuela*

* Specimens housed in UF collection.

### Key to Calcaritermes in the UF collection based on the soldier or pseudergate caste

**Table:** 

1	Pseudergates (pseudergates without large wing buds) lack mesonotal rasp or concave posterior margin of pronotum; soldier with frontal furrow rather even in depth extending length of frons at a shallow angle, frontal lobes with distinct elongate rugosity (Fig. 3G)	*Calcaritermes temnocephalus*
–	Pseudergates with mesonotal rasp and concave posterior margin of pronotum (Fig. 4C), soldier unlike above	2
2	Soldier maximum head width 1.4 mm or more, head capsule elongate; furrow rugose; frontal lobes clearly not overhanging frons (Fig. 3A)	*Calcaritermes guatemalae*
–	Soldier maximum head width less, or much less than 1.4 mm, head variable	3
3	Head capsule somewhat to clearly elongate (Figs 3C, 3D, 3E, 3H)	4
–	Head capsule truncate, mandibles short (Figs 3B, 3F)	7
4	Frontal furrow unsculptured; frontal lobes smooth; lobes obtuse in angle ca. 145° (Fig. 3C)	*Calcaritermes nigriceps*
–	Frontal furrow and lobes with sculpturing; lobes form angle ca. 90–100° (Figs 3D, 3E, 3H)	5
5	Frontal lobes rounded, ovoid depression near center of lobes (Fig. 3H)	*Calcaritermes snyderi*
–	Frontal lobes more acutely pointed, no ovoid depression near center of lobes (Figs 3D, 3E)	6
6	Larger species, maximum head width 1.2–1.3 mm, frontal lobes with slightly overhanging tips, neotropical distribution (Fig. 3E)	*Calcaritermes emarginicollis*
–	Smaller species, maximum head width 1.1 mm, frontal lobes evenly angled, Nearctic distribution only (Fig. 3D)	*Calcaritermes nearcticus*
7	Frontal lobes nearly overhanging frons; raised well above vertex; distinct ovoid depression in center of lobes (Fig. 3F)	*Calcaritermes brevicollis*
–	Frontal lobes not overhanging frons; almost even with vertex; lacking ovoid depression in center of lobes (Fig. 3B)	*Calcaritermes rioensis*

### Mesonotum morphology

Mandible dentition of pseudergate or nymphal castes has been used for generic grouping of some Kalotermitidae (Krishna 1961), but for most genera, these weak and often overlapping characters by themselves lead to tenuous or uncertain identifications. In describing the imago and immature forms of *Calcaritermes emarginicollis* from Costa Rica, Snyder (1925a) was first to observe and depict (Snyder’s Figs 2, 4) that the mesonotum of the brachypterous nymph had an “aspirate or rugose area” while in the presoldier caste, he noted that the aspirate area of the mesonotum was elevated. (Snyder (1925b, 1926a, b), Light (1933), and Krishna (1962) described eight more *Calcaritermes* species, but the mesonotal rugosity was not mentioned again for any caste until Miller (1943) reported that nymphs of *Calcaritermes nearcticus* had a “slightly raised median mesonotal area upon which appear numerous aspirities”. In Krishna’s 1961 revision of the Kalotermitidae, the mesonotal character was not mentioned.

These mesonotal “rasps” were found on all apterous pseudergates, early stage brachypterous nymphs, and most soldiers of *Calcaritermes* for species in the UF Collection (Table 1) with the exception of *Calcaritermes temnocephalus* in which the rasp is absent. Under magnification, it was observed that each of these rasps actually consist of a single layer of slightly overlapping spatulate scales with basal attachments at their anterior ends (Figs 4A, 4B, 4D). The mesonotal rasps have a midline divide and form an elevated mound raised above the remainder of the dorsum (Fig. 4C, right). The posterior margin of the pronotum of all eragtoid/nymphoid castes, except again *Calcaritermes temnocephalus*, has a posterior marginal concavity that partially surrounds the anterior of the rasp (Fig. 4C, arrow). The pronotum is steeply angled toward the head anterior to the rasp (Fig. 4C, right). The scale patterns and lateral profile of the rasps vary somewhat among species (e.g., Figs 4A, 4B) but no species-specific morphology was investigated in this study. No rasp was found on any mature reproductive and the robustness of the rasp was inversely proportional to wing bud size disappearing when the nymphs were one molt from adulthood. The mesonotal rasp is the first external character to provide a diagnostic, generic identification of an immature kalotermitid.

Microscopic examination of the mesonotal rasps from ethanol-preserved specimens did not reveal microbial material around the scales. However, when live specimens of *Calcaritermes nearcticus* were prepared for SEM without cleaning or rinsing, an organic (microbial?) paste was observed between the scales (Fig 4D).

**Queen physogastry.** Over the years, I have observed hundreds of mature queens in kalotermitid nests but was struck by the extreme queen physogastry in *Calcaritermes temnocephalus*. On 26 May 2008, two colonies of *Calcaritermes temnocephalus* were collected by the UF survey team at Silva Seco de Capadare, Guiermo, Venezuela (11.154, -68.590, elev. 58m). Both colonies were large and occupied rather sound wood from which a mature primary queen was removed (Fig. 4E). The extent of physogastry of these queens is what is typically observed in the Rhinotermitidae or Termitidae in which the intersegmental membrane stretches well beyond the width of the tergites or sternites. Typically the extended intersegmental membrane in primary queens of the Kalotermitidae is narrower than the width of adjacent abdominal sclerites, but in the *Calcaritermes temnocephalus* queens, the membrane is much wider than the sclerites. Eggs from one of the *Calcaritermes temnocephalus* colonies (Fig. 4E, arrow) and from a *Calcaritermes brevicollis* colony in Panama (Fig. 4F, arrows) also appeared disproportionally large compared to other kalotermitids.

**Nests.** *Calcaritermes* colonies infest damp or wet wood, usually in the shade of forest canopy. At ground level, populations are never plentiful in a given area. However, Roisin et al. (2006) found that the preponderance of *Calcaritermes brevicollis* colonies in a Panamanian rain forest were occupying dead branches 10 m or higher above the ground. Workers and soldiers move rather slowly compared to most other kalotermitids, but in contrast, the alates flutter in hyperkinetic fashion as soon as their galleries are opened. Bourguignon et al. (2009) collected all dispersing *Calcaritermes brevicollis* alates during March to June in flight intercept traps. No alates were attracted to light traps indicating that *Calcaritermes brevicollis*, and probably the other dark-colored species, are daytime flyers.

The gallery system of *Calcaritermes* differs from other kalotermitids in several distinct ways. First, the galleries are narrow and tubular, maybe allowing only two termites to pass at one time. The galleries are spaced rather far apart in the wood matrix, thus occupying a relative small volume of the colonized member (Figs 5A, 5B). Secondly, the galleries contain very few loose fecal pellets, but gallery surfaces are generously lined with what appears to be a moist fecal/microbial? paste (Figs 5A, 5B, 5C). Miller (1949) noted that *Calcaritermes nearcticus* “lines some of its galleries with a coating of brownish material”. Thirdly, the peripheries of the galleries are stained or exhibit halos suggesting fungal infection emanating from the gallery surfaces into the wood at varying depths (Figs 5A, 5B). Again, the exception is *Calcaritermes temnocephalus* which infests wood in open, often dryer conditions, had less fecal coating and microbial growth (Fig. 5C) in their galleries.

Given the mesonotal rasp, the low volume of wood excavated, gallery coating, and peripheral gallery staining, one can hypothesize that *Calcaritermes* derives some nutrition via a symbiotic relationship with microbes growing on the surface of their galleries. The rasp may be used by foragers to inoculate gallery surfaces with fungal or bacterial spores analogous to the mycangium (Stone et al. 2007) found in bark beetles (Scolytinae). Unlike the mycangia of adult beetles, *Calcaritermes* adults (alates) show no obvious external structure for horizontal transfer of spores to new nesting sites although alimentary storage is a possibility. So whether *Calcaritermes*, like bark beetles, have some form of external association with microorganism (Gilbertson 1984) or actually rely on symbiotic mycophagy (Harrington 2005) remains to be studied.
